# A novel mammarenavirus (family *Arenaviridae*) in hedgehogs (*Erinaceus roumanicus*) in Europe

**DOI:** 10.1007/s00705-023-05804-8

**Published:** 2023-06-08

**Authors:** Gábor Reuter, Ákos Boros, Károly Takáts, Róbert Mátics, Péter Pankovics

**Affiliations:** 1 Department of Medical Microbiology and Immunology, Medical School, University of Pécs, Szigeti út 12., 7624 Pécs, Hungary; 2 Hungarian Nature Research Society, Ajka, Hungary

## Abstract

In this study, a novel mammarenavirus (family *Arenaviridae*) was identified in a hedgehog (family Erinaceidae) in Hungary and genetically characterized. Mecsek Mountains virus (MEMV, OP191655, OP191656) was detected in nine (45%) out of 20 faecal specimens collected from a Northern white-breasted hedgehog (*Erinaceus roumanicus*). The L-segment proteins (RdRp and Z) and S-segment proteins (NP and GPC) of MEMV had 67.5%/70% and 74.6%/65.6% amino acid sequence identity, respectively, to the corresponding proteins of Alxa virus (species *Mammarenavirus alashanense*) identified recently in an anal swab from a three-toed jerboa (*Dipus sagitta*) in China. MEMV is the second known arenavirus endemic in Europe.

**Supplementary Information:**

The online version contains supplementary material available at 10.1007/s00705-023-05804-8.

Arenaviruses (family *Arenaviridae*) are enveloped viruses with genomes consisting of two or three single-stranded RNA segments totaling about 10.5 kb. The family is currently divided into five genera (*Antennavirus*, *Hartmanivirus*, *Innmovirus*, *Mammarenavirus*, and *Reptarenavirus*) and 60 species [1, 2]. The known host range of arenaviruses predominantly includes small mammals (mammarenaviruses), especially rodents (mice, rats, jerboas), bats, and ticks as well as non-traditional hosts such as snakes (hartmaniviruses and reptarenaviruses) and fish (antennaviruses) [2]. Each arenavirus is usually associated with a particular host species in which it is maintained. However, arenaviruses probably are not as host-specific as previously suggested, and additional animals may serve as natural reservoirs [3]. Arenaviruses are capable of causing chronic infections in their hosts. The infectious virus is present in the blood (viremia) and is also secreted into body fluids (saliva, urine, semen) and faeces, which is presumably the source of zoonotic human infections [4]. The rodent-borne lymphocytic choriomeningitis virus (LCMV) is the only known arenavirus endemic in Europe [5]. This neglected zoonotic mammarenavirus is able to cause central nervous system and intrauterine infections and diseases in humans [5].

The mammarenavirus virion contains two ambisense RNA segments and expresses four viral proteins. The L-segment encodes the RNA-dependent RNA polymerase (RdRp) and the zinc-binding matrix protein (Z); the S-segment encodes the nucleoprotein (NP) and the glycoprotein precursor (GPC). The non-overlapping open reading frames (ORFs) of opposite polarities are separated by a non-coding intergenic region (IGR) in both segments [5]. Mammarenaviruses can be divided into two serogroups based on their (phylo)genetic diversity and geographic distribution: Old Word complex viruses in the Eastern Hemisphere (Europe, Asia, and Africa) and New Word complex viruses in the Western Hemisphere (America). Only LCMV is known to have worldwide distribution [5].

Hedgehogs (family Erinaceidae) are small omnivorous mammals. There are 17 species living in Europe, Asia, Africa, and New Zealand; however, they have become popular in recent years as pets, especially in North America [6]. Hedgehogs are not known to be hosts of any arenaviruses.

In this study, faecal samples from hedgehogs were investigated for the potential presence of LCMV by RT-PCR. We report the serendipitous detection and complete genome sequence of a novel mammarenavirus from hedgehogs in Europe.

A total of 20 faecal specimens were collected from hedgehogs [7] were identified as Northern white-breasted hedgehogs (*Erinaceus roumanicus*) using a PCR and Sanger sequencing method with the in-house-designed primers Hedgehog-cytochrome-b-F/R (F, 5’-GAGGCGCTACAGTCATTACTA-3’; R, 5’-CATTGACTTACAGGTCGGAAT-3’). Eight samples were collected from carcasses of road-hit hedgehogs in 2015, from the Hungarian capital Budapest and its suburbs. Twelve faecal samples (MR1-MR9 and MR11-MR13) were collected from hedgehogs in 2015 in southwestern Hungary. These adult hedgehogs were captured at two different locations: in downtown Pécs (MR11-13; geolocation lat. 46.0704, long. 18.2197) and on the confines of the city (MR1-9: geolocation lat. 46.1197, long. 18.3033) from natural wild areas (Mecsek Mountains), 8.44 air kilometers apart from each other, so they most probably belonged to separate colony groups. Samples were collected by qualified biologists with valid permission from the National Inspectorate for Environment, Nature and Water (4018-4/2015). The collected faecal samples were stored at -80°C.

Viral RNA was isolated from faecal samples using TRIzol Reagent (Thermo Fisher Scientific, Waltham, MA, USA) according to the manufacturer’s instructions. Specimens were tested by semi-nested RT-PCR using screening primers designed for the LCMV L-protein (RdRp) (LCMV-F1, 5’-CANTGGCAYATGCAYAA-3’; LCMV-F2, 5’-AGYCTHATTGAYATGGG-3’; LCMV-R, 5’-ACYTCYTCNCCCCANACATA-3’) yielding a 320-bp-long inner amplicon. The complete arenavirus genome was amplified and determined using primers designed based on sequences of the closest arenavirus relatives and by the “primer walking” method. Briefly, a two-step RT-PCR method was used. A cDNA transcript from the RNA was prepared using Maxima H Minus RT (Thermo Fisher Scientific) according to the manufacturer’s instructions. The incubation time was 55 minutes, and the reaction temperature was varied between 50 and 55°C according to the predicted secondary structure of the RNA. Removal of RNA after first-strand cDNA synthesis was carried out using RNase H (Thermo Fisher Scientific) for 20 minutes at 37°C. Based on the length of the predicted PCR products, DreamTaq DNA Polymerase (Thermo Fisher Scientific) or Phusion™ High-Fidelity DNA Polymerase (Thermo Fisher Scientific) was used in the PCR reactions according to the manufacturer's instructions. The PCR conditions were modified based on the manufacturer’s description of the enzymes and the melting temperatures of the primers used. The terminal sequence of the L segment was determined using the 5’ RACE method, with modifications. The RNA of the L-segment was transcribed to cDNA using a sequence-specific oligonucleotide and Maxima H Minus RT. The cDNA was then treated with RNase H and purified using a GeneJET PCR Purification Kit (Thermo Fisher Scientific). An adapter (5’-/5Phos/TTTTTTAACTGATCACCTCTAGACC/3InvdT/-3’) was ligated to the 3’ end of the cDNA using T4 RNA ligase (10 U/µL; Thermo Fisher Scientific) [8]. The PCR reaction was carried out using sequence-specific primers (Adapter-Anchor, 5’-GGTCTAGAGGTGATCAGTTA-3’; L-6657-F, 5’-CTAGTCTTGGTGCATTCCAATT-3’). The PCR product was sequenced directly using the sequence-specific primer L-6657-F. The end of the other L-segment and both termini of the S-segment were amplified and sequenced using reverse-complementary primers. Newly designed common arenavirus semi-nested RT-PCR screening primers (univ-F1, 5’-TCTTATAARGANCANGTKGG-3’; univ-F2, 5’-AAATGGGGNCCNATGATGT-3’; univ-R, 5’-ATCTGATCATCACTNGATGT-3’) were designed based on the nucleotide sequences of the L-segments of LCMV (AY847351), Alxa virus (KY432892), and the study virus (OP191655), yielding 632- to 635-bp-long outer and 419- to 422-bp-long inner amplicons, respectively. PCR products were sequenced directly by the Sanger method using a BigDye Terminator v1.1 Cycle Sequencing Kit (Thermo Fisher Scientific) and then run on an automated sequencer (AB3500 Genetic Analyzer, Applied Biosystems, Hitachi, Tokyo, Japan).

The complete nucleotide (and viral protein) sequences of the L and S genome segments of Mecsek Mountains virus MR1 and five other arenavirus S genome segments (MR2, MR3, MR7, MR8, and MR9) were submitted to the GenBank database under accession numbers OP191655, OP191656, and OQ543564-OQ543568, respectively.

Using the initial LCMV screening primers, five (25%) faecal specimens (MR1, MR2, MR4, MR7, and MR9) collected in the Mecsek Mountains yielded PCR products of the expected sizes. Sequencing revealed that these amplicons represented arenavirus sequences with the closest match (60% nt and 77% aa identity) to the RdRp protein of Alxa virus (species *Mammarenavirus alashanense*, genus *Mammarenavirus;* GenBank no. KY432892). Alxa virus was identified in 2018 in anal swabs collected from three-toed jerboas (*Dipus sagitta,* family Dipodidae) in Inner Mongolia Autonomous Region, China [9]. Specimen MR1 was selected for complete genome sequencing, and this virus was named “Mecsek Mountains virus” (MEMV).

The complete L-segment of the MEMV genome (OP191655) is 7,393 nt long. It has 70% nt sequence identity to the L-segment of Alxa virus (KY432892; query coverage, 89%) as the closest match in GenBank. The RdRp protein is 2,241 aa (6,726 nt) long. It has 67.5% and 45.7% aa sequence identity to the corresponding protein of Alxa virus (KY432892, query coverage, 100%) and rat mammarenavirus (MG736231; query coverage, 99%), respectively. The N-terminus of the L protein (NL1 domain) contains the P_88_D…(E_102_)K_115_K_122_ type-II RNA endonuclease motif [10]. The Z protein is 91 aa (276 nt) long. It has 70% and 62.3% aa sequence identity to the corresponding protein of Alxa virus (KY432892; query coverage, 95%) and Lassa virus (KM821846; query coverage, 95%), respectively. It contains all of the conserved aa motifs (**G**_2_N**K**PT**K**LESNRRL_14_, N-terminal myristoylation site, zinc-binding RING domain, **Y**_50_LC**L**, **Y**_70_CP**L**, and C-terminal **P**_83_**SAPPPY**_89_) characteristic of mammarenaviruses [11].

The complete S-segment of the MEMV genome (OP191656) is 3,536 nt long. It has 71% nt sequence identity to the S-segment of Alxa virus (KY432893; query coverage, 89%). The NP is 607 aa (1,824 nt) and is therefore longer than the other known arenavirus NPs (557 aa-593 aa), with a 37-aa-long extension in its N-terminal region (aa positions 5 to 41). The NP has 74.6% and 59.2% aa sequence identity to the corresponding proteins of Alxa virus (KY432893; query coverage, 93%) and Luna mammarenavirus (NC_016152; query coverage, 93%), respectively. The GPC is 487 aa (1,464 nt) long, and it has 65.6% and 56.1% aa sequence identity to the corresponding proteins of Alxa virus (KY432893; query coverage, 100%) and Loei River mammarenavirus (NC_038364; query coverage, 100%). The aa sequence motif at the GP1/GP2 cleavage site [12] is SNRVIT**R**_252_R**L**QALFKWSLTDS.

In the L-segment, the 5’, IGR, and 3’ untranslated regions are 78 nt, 216 nt, and 97 nt long, respectively, and in the S-segment, they are 71 nt, 96 nt, and 81 nt long, respectively. The four termini of the RNA segment ends have identical, inverted complementary nucleotide sequences (UGCACAGGGGAUCCUAGGC … / … GCCUAGGAUCCCCUGUGCA).

Phylogenetic analysis based on the full-length aa sequences of the RdRp (L-segment) and NP (S-segment) showed that MEMV forms a distinct lineage with Alxa virus, together with arenaviruses of the Old World complex (Fig. 1). The phylogenetic trees suggest that Alxa virus and MEMV have evolved separately and are distinct from all other *Murinae*-borne arenaviruses of the Old World complex [9].

**Fig. 1:**
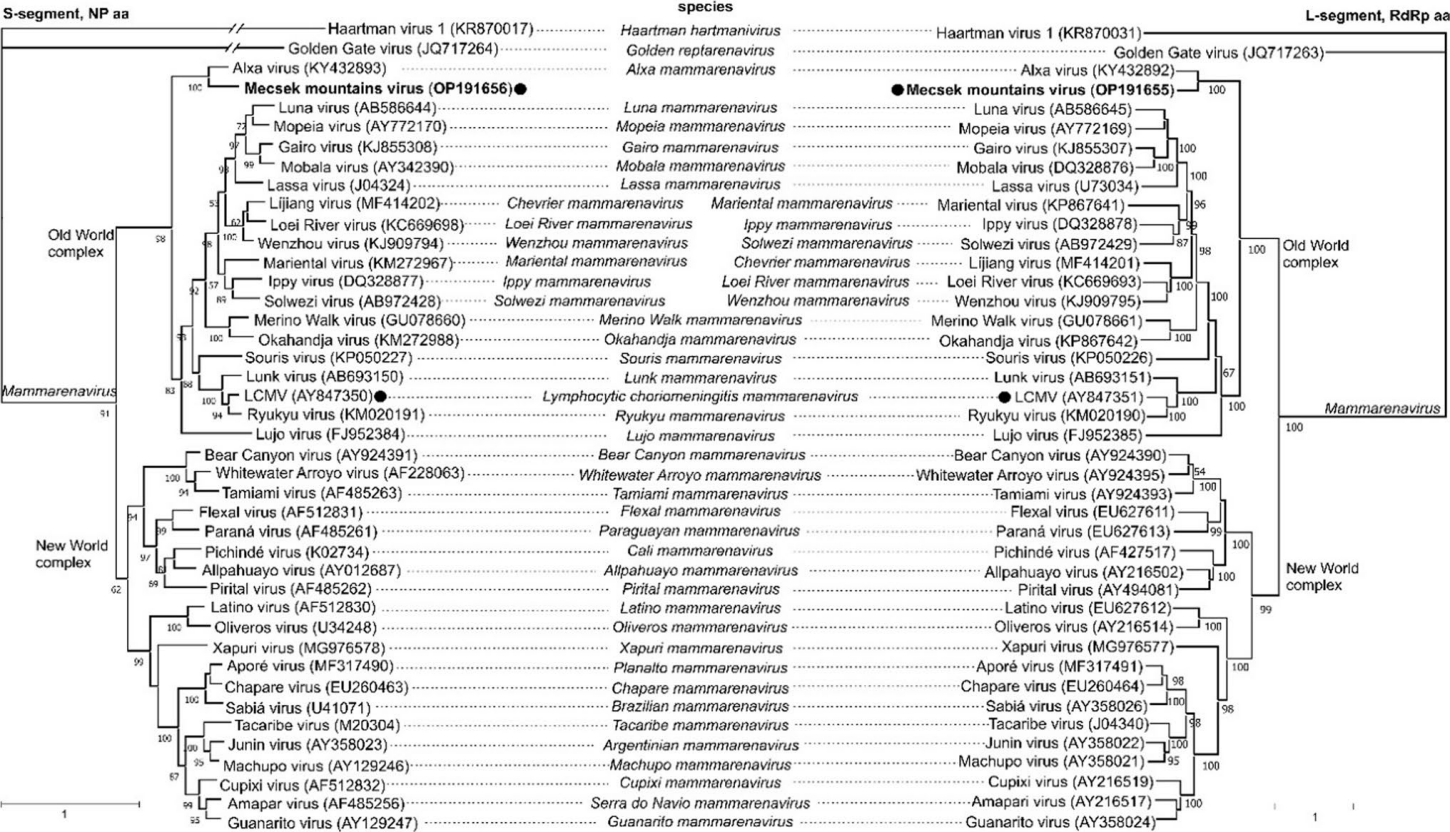
Maximum-likelihood phylogenetic trees generated from webPRANK alignments [13] of the complete RdRp (S-segment, left) and NP (L-segment, right) amino acid sequences of mammarenaviruses using the best-fit model of protein evolution (LG+G) selected previously [2]. Maximum-likelihood trees with 1,000 ultrafast bootstrap replicates were produced using the IQ-Tree web server [14] and visualized using MEGA X [15] and Corel Draw Standard 2020. Mammarenaviruses can be divided into two serogroups: Old Word complex viruses in the Eastern Hemisphere (Europe, Asia, and Africa) and New World complex viruses in the Western Hemisphere (America). Mecsek Mountains virus is shown in bold. Dark circles indicate the known mammarenaviruses in Europe. LCMV: lymphocytic choriomeningitis virus

Using the re-designed common primer pairs (univ-F1/F2/R) nine (45%) of the 20 faecal specimens (MR1-MR9) were positive for MEMV by semi-nested RT-PCR, all of which were from the Mecsek Mountains area. Sequencing of these amplicons (383 bp) showed that they had 96-100% nt sequence identity (100% aa sequence identity) to each other, representing two groups of nucleotide sequence variants with six (MR1, MR2, MR4, MR6, MR8 and MR9) and three (MR3, MR5 and MR7) identical strains. Based on the complete, 3,536-nucleotide-long S genome segments of six MEMV strains (MR1, OP191656; MR2, OQ543564; MR3, OQ543565; MR7, OQ543566; MR8, OQ543567 and MR9, OQ543568), the nucleotide sequence identity values are between 95% and 99%.

Consistent with the ICTV *Arenaviridae* Study Group species demarcation criteria [12], MEMV has less than 80% and 76% nt sequence identity in the S- and L-segments, respectively, and less than 88% aa sequence identity in the NP to the most closely related member of the genus, Alxa virus. MEMV therefore potentially represents a novel arenavirus species in the genus *Mammarenavirus*. This novel virus is associated with hedgehog, which has not previously been known to be a host of any arenaviruses. In addition, except for LCMV, the endemic presence of other arenaviruses has not been known in Europe to date.

The presence of mammarenaviruses in non-rodents is rare, only Tacaribe and Alxa viruses are known [9]. MEMV was detected serendipitously in nearly half of the faecal specimens collected from Northern white-breasted hedgehogs (*Erinaceus roumanicus*); however, it was present specifically in the specimens collected from wild-living hedgehogs in natural wild areas of the Mecsek Mountains. This finding indicates that mammarenaviruses are present in more mammalian host species than previously thought and that *Murinae* species are not their only hosts. The common arenavirus semi-nested RT-PCR screening primers (univ-F1/F2/R) that were designed based on LCMV, Alxa virus, and MEMV sequences in this study could be suitable for molecular epidemiological studies and discovery of novel arenaviruses. Using this method, MEMV mammarenavirus sequences were not found in our archived animal specimen bank collected from birds of prey (kestrel and red-footed falcon) [16] and rodents (wild and laboratory mice and rats) [17].

Wild animals and exotic pets such as hedgehogs pose a risk of zoonotic transmission of pathogens, including viruses [18]. The results of this study expand our knowledge of both the genetic diversity of arenaviruses and the spectrum of their host species in Europe, where the presence of arenaviruses has not been well studied.

## Supplementary Information

Below is the link to the electronic supplementary material.

Supplementary file1 (TXT 39 KB)

## Data Availability

Not applicable.
